# Whole-heart four-dimensional flow can be acquired with preserved quality without respiratory gating, facilitating clinical use: a head-to-head comparison

**DOI:** 10.1186/s12880-015-0061-4

**Published:** 2015-06-18

**Authors:** Mikael Kanski, Johannes Töger, Katarina Steding-Ehrenborg, Christos Xanthis, Karin Markenroth Bloch, Einar Heiberg, Marcus Carlsson, Håkan Arheden

**Affiliations:** Department of Clinical Physiology, Lund University, Lund University Hospital, Lund, Sweden; Department of Numerical Analysis, Center of Mathematical Sciences, Lund University, Lund, Sweden; Department of Computer Science and Biomedical informatics, University of Thessaly, Lamia, Greece; Philips Healthcare, Lund, Sweden; Department of Biomedical Engineering, Faculty of Engineering, Lund University, Lund, Sweden; Center for Mathematics, Faculty of Engineering, Lund University, Lund, Sweden

**Keywords:** Cardiac MRI, 4D flow, Respiratory gating, Kinetic energy, Particle trace, Vortex ring size

## Abstract

**Background:**

Respiratory gating is often used in 4D-flow acquisition to reduce motion artifacts. However, gating increases scan time. The aim of this study was to investigate if respiratory gating can be excluded from 4D flow acquisitions without affecting quantitative intracardiac parameters.

**Methods:**

Eight volunteers underwent CMR at 1.5 T with a 5-channel coil (5ch). Imaging included 2D flow measurements and whole-heart 4D flow with and without respiratory gating (Resp(+), Resp(−)). Stroke volume (SV), particle-trace volumes, kinetic energy, and vortex-ring volume were obtained from 4D flow-data. These parameters were compared between 5ch Resp(+) and 5ch Resp(−). In addition, 20 patients with heart failure were scanned using a 32-channel coil (32ch), and particle-trace volumes were compared to planimetric SV. Paired comparisons were performed using Wilcoxon’s test and correlation analysis using Pearson r. Agreement was assessed as bias ± SD.

**Results:**

Stroke volume from 4D flow was lower compared to 2D flow both with and without respiratory gating (5ch Resp(+) 88 ± 18 vs 97 ± 24.0, *p* = 0.001; 5ch Resp(−) 86 ± 16 vs 97.1 ± 22.7, *p* < 0.01). There was a good correlation between Resp(+) and Resp(−) for particle-trace derived volumes (R^2^ = 0.82, 0.2 ± 9.4 ml), mean kinetic energy (R^2^ = 0.86, 0.07 ± 0.21 mJ), peak kinetic energy (R^2^ = 0.88, 0.14 ± 0.77 mJ), and vortex-ring volume (R^2^ = 0.70, −2.5 ± 9.4 ml). Furthermore, good correlation was found between particle-trace volume and planimetric SV in patients for 32ch Resp(−) (R^2^ = 0.62, −4.2 ± 17.6 ml) and in healthy volunteers for 5ch Resp(+) (R^2^ = 0.89, −11 ± 7 ml), and 5ch Resp(−) (R^2^ = 0.93, −7.5 ± 5.4 ml), Average scan duration for Resp(−) was shorter compared to Resp(+) (27 ± 9 min vs 61 ± 19 min, *p* < 0.05).

**Conclusions:**

Whole-heart 4D flow can be acquired with preserved quantitative results without respiratory gating, facilitating clinical use.

**Electronic supplementary material:**

The online version of this article (doi:10.1186/s12880-015-0061-4) contains supplementary material, which is available to authorized users.

## Background

The use of three-dimensional, time-resolved, three-component (4D) phase-contrast cardiovascular magnetic resonance (CMR), also called 4D flow, is increasingly used to study blood flow patterns [1-6], quantify kinetic energy [4, 7-11], and for particle tracing analysis [5, 12]. Due to the possibility of off-line flow quantifications, visualization of vascular anatomy and derivation of various parameters, 4D flow is emerging as a new powerful tool in CMR, e.g. in patients with congenital vascular anomalies [13, 14]. However, 4D flow of the whole heart is limited by scan times [5], which are long even when applying acceleration techniques [15]. Respiratory gating, usually being used to reduce respiratory motion artifacts [16-19], further prolongs the acquisition time by unpredictable amounts, whereas it may be difficult in some patients. Acquisition of 4D flow without respiratory gating has the advantage of reduced and known scan time and could therefore simplify 4D flow acquisitions in the clinical setting in orthopnoeic and claustrophobic patients.

Nordmeyer et al. [20] have previously investigated the effect of respiratory gating in 4D flow and found a large reduction in scan time for 4D flow without respiratory gating compared to with respiratory gating, and no significant differences for stroke volume (SV) quantifications in greater vessels. However, intracardiac 4D flow was not investigated. Intracardiac measures, especially those based on particle tracing, may be more sensitive to respiratory motion as the diaphragm movement affects the position of the heart, and the effect of respiratory gating on intracardiac 4D flow parameters has not previously been investigated. Therefore, we aimed to investigate the effect on quantitative intracardiac flow parameters and particle tracing quality in 4D flow with and without respiratory gating.

## Material and methods

### Study design and CMR protocol

The study was approved by the Regional Ethical Review Board in Lund, Sweden, and all subjects provided written consent. Ten (10) healthy volunteers (4 men, 6 women, age 27 ± 2 years) underwent CMR in a 1.5 T Philips Achieva scanner (Philips Healthcare, Best, The Netherlands). The CMR protocol consisted of cardiac function cine imaging (short axis, 2–3- and 4-chamber view), 2D flow measurements (aorta and main pulmonary artery (MPA)) and 4D flow sequences with respiratory gating (Resp(+)) and without any respiratory gating (Resp(−)). The protocol was repeated twice for each subject on the same day; the first scan using a 32 channel coil (32ch), and the second scan using a 5 channel coil (5ch). In 2 of the 10 subjects, the 5ch and 32ch scans could not be performed on the same day and were performed after 7 days in one subject. The other subject was re-scanned after 36 days but the dataset was excluded from comparison between coils as it was deemed too long time between 32ch and 5ch scans. Due to artifacts seen in 4D flow Resp(+) acquisitions using the 32ch coil (unpublished observations; probably due to steady-state loss or residual breathing), the 32ch scan only included the Resp(−) 4D flow sequence. One possible explanation to why these artifacts are seen in the 32ch acquisition and not the 5ch acquisition is the fact that the 32ch coil consists of smaller coils and therefore signal from the subcutaneous fat will be more prominent compared to the 5ch coil. The 4D flow sequences were both preceded and followed by 2D flow acquisitions in order to test intrastudy variability in stroke volume (SV). The CMR protocol is summarized in Table 1.

**Table 1:** CMR protocol for healthy volunteers. Ao = Aorta, MPA = Main pulmonary artery, 2ch, 3ch, 4ch = two-chamber, three-chamber, four-chamber

Sequence	
1.	Subject placed in scanner with 32-channel cardiac coil
2.	Cine images: 2ch, 3ch, 4ch, short-axis
3.	2D flow: Ao, MPA
4.	4D flow Resp(−)
5.	2D flow: Ao, MPA
6.	Switch to 5-channel cardiac coil, short break for subject
7.	Cine images: 2ch, 3ch, 4ch, short-axis
8.	2D flow: Ao, MPA
9.	4D flow Resp(+)
10.	4D flow Resp(−)
11.	2D flow: Ao, MPA
12.	End of protocol

Furthermore, 20 patients with heart failure underwent CMR using a 32ch coil including cardiac function cine imaging (short axis, 2–3- and 4-chamber view), and Resp(−) 4D flow.

### CMR sequence parameters

#### 4D flow

A segmented gradient echo (TFE) sequence, 2 views per segment, with retrospective ECG-triggering and a SENSE parallel imaging factor of 2 was used. 4D flow was acquired with respiratory gating (Resp(+)) and without respiratory gating (Resp(−)) with a field of view covering the entire heart. In the Resp(+) sequence, a pencil navigator beam was positioned in the center of the right lung diaphragm with two-thirds of the navigator in the liver. The width of the navigator acceptance window was 5 mm. The acquired temporal resolution was 51–54 ms, i.e. 15–22 phases acquired, which were then reconstructed to 40 heart phases. Typical scanning parameters were TE/TR: 3.7/6.2 ms, flip angle: 8°, acquired and reconstructed voxel size: 3 × 3 × 3 mm^3^, number of acquired slices 40–52, VENC 100 cm/s. Concomitant gradient terms were compensated by the CMR scanner. Phase unwrapping was performed offline using an automatic algorithm with manual corrections as needed. Phase background errors (e.g. due to eddy currents) were corrected offline by subtraction of a linear fit of velocities in stationary tissue.

#### 2D flow

A non-segmented phase-contrast gradient echo sequence with retrospective ECG-triggering during free breathing was used to measure through-plane flow. Typical scanning parameters were TE/TR: 5.3/8.6 ms, flip angle: 15°, temporal resolution: 23–35 ms (35 phases), spatial resolution: 1.2 × 1.2 × 6 mm, VENC: 200 cm/s for both aorta (Ao) and main pulmonary artery (MPA). For 2D flow scans, phase background as well as concomitant gradients were compensated by the scanner software.

#### Cine images

Standard steady-state free precession cine images were acquired in the two-chamber, three-chamber, four-chamber and short axis views. Typical scanning parameters were: TE/TR: 1.6/3.2 ms, flip angle: 60°, spatial resolution: 1.25 × 1.25 × 8 mm, no slice gap.

### Particle tracing

Particle tracing (PT) uses the measured 4D flow information to compute blood motion. The PT quality of the collected 4D flow data was evaluated as follows, based on a method previously described [5]:

1. The position of the short-axis cine images were manually adjusted to match the 4D flow data to reduce offsets due to breath-holding positions, as shown in Fig. 1.

**Fig. 1:**
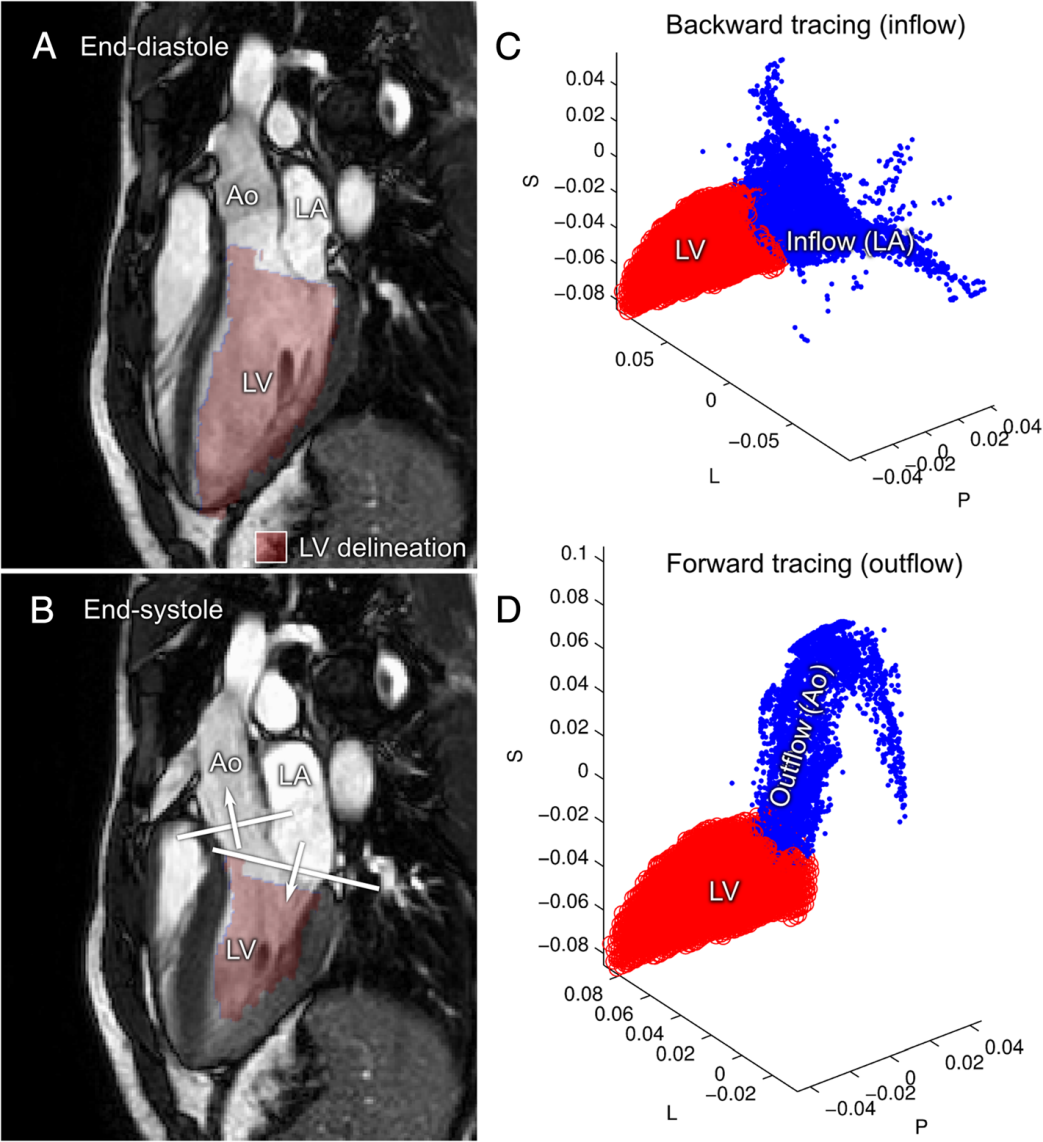
Data evaluation of particle trace (PT). **a**: Using the short-axis manual delineation of the LV, all 4D flow voxels inside the LV at end-diastole were selected for particle trace analysis. Panel B shows the planes used to define inflow and outflow. Panel **c** and **d**: One particle was placed in each voxel and traced backward in time to end-systole (*inflow*, **c**), and forward to end-systole (*outflow*, **d**). For inflow analysis **c**, all particles on the basal side of the mitral valve at end-systole (**b**) were considered as inflowing blood. For outflow analysis (**d**), all particles on the basal side of the aortic valve (**b**) at end-systole were considered as inflowing blood. *L = Left-Right axis, P = Posterior-Anterior axis, S = Superior-Inferior axis*
2. The volume of the left ventricle (LV) was manually delineated at end-diastole, as seen in Fig. 1. Furthermore, the locations of the mitral valve and aortic valve at end-systole were manually delineated using long-axis (2ch, 3ch, 4ch) cine images.
3. One particle for each 4D flow voxel in the LV end-diastolic volume was traced backward in time to end-systole to find the PT inflow volume, and traced forward in time to end-systole to find the PT outflow volume.
4. Particles traced forward in time with a final position on the basal side of the aortic valve were considered as outflowing blood. Particles traced backward in time with a final position on the basal side of the mitral valve were considered as inflowing blood. Each particle contributed a volume equal to the 4D flow voxel size.
5. In healthy volunteers with normal valve function, the particle trace inflow and outflow should be equal. Therefore, the inflowing volume was compared to the outflowing volume.
6. Fig. 1 shows an illustration of the above method. Particle tracing was performed using a 4th-order Runge–Kutta method with a constant timestep of 5 ms. Linear interpolation of the 4D flow velocity data was used in space and time.

### Image analysis

The freely available software Segment (http://segment.heiberg.se) was used for all image analysis [21]. Particle tracing was performed using an in-house developed code to Segment, consisting of a 4th Runge–Kutta method and linear interpolation in space and time, as previously described [12].

#### Stroke Volume analysis

In short, the stroke volume (SV) was first measured in the 2D flow images by delineating the vessels over time using a region of interest (ROI). In the 4D flow data, a 2D plane was computed from the 2D flow images, and the ROI was copied to the computed image plane. Manual corrections of the ROI position were performed if needed.

#### Kinetic Energy analysis

The LV was defined by manually drawing the contours of the blood volume in short-axis slices over the entire cardiac cycle. The delineations were then transferred to the 4D data set and KE was calculated as the sum of ½mv^2^ for each voxel [22], where m is the mass of blood in one voxel and v is the velocity in the voxel. Manual corrections of delineations were performed when needed.

#### Vortex ring volume

Lagrangian coherent structures (LCS) were computed as previously described [12]. Particle tracing computations required for the LCS algorithm were implemented in CUDA-C and performed on Graphical Processing Unit (GPU) cards. LCS indicative of vortex-ring formation were delineated in the long-axis views. Then, the vortex-ring volume was delineated in short-axis slices 4 mm apart, guided by the long-axis delineations. Total vortex volume was defined as the summed volume of all slices.

### Particle trace visualizations

Blood flow was visualized using FourFlow v1.2.14, open source software for flow visualization (http://fourflow.heiberg.se). Particles were emitted every 25–30 ms during diastole in the left atrium to visualize diastolic inflow into the LV. Images were saved after the rapid filling phase of the LV.

### Statistical analysis

Statistical analysis was performed using Graphpad for Windows (v6.04, Graphpad Software Inc, La Jolla, USA). Group values are given as mean ± SD. Measurements acquired from different sequences were compared using a paired non-parametric two-tailed test (Wilcoxon) and linear regression. Correlation analysis was performed using Pearson r. Agreement was assessed as bias ± SD [23]. Differences with *P*-values <0.05 were considered statistically significant.

## Results

Subject and patient characteristics are shown in Table 2.

**Table 2:** Subject demographics and characteristics

	Healthy volunteers	Patients with heart failure
Sex (male/female, n)	5/3	16/4
Age (years)	28 ± 1	68 ± 8
Height (cm)	172 ± 11	176 ± 9
Heart rate (bpm)	66 ± 10	68 ± 16
LV-EF (%)	60 ± 4	28 ± 10
Underlying disease (%)		
- Dilated cardiomyopathy	N/A	45
- Ischemic heart disease	N/A	55

### Excluded data

In one subject, the difference in scan duration between Resp(−) and Resp(+) 4D flow acquisitions was only 2 min (5 %), suggesting inaccurate navigator triggering, and these data were therefore excluded. Another subject was excluded due to a zipper artifact through the base of left ventricle, however, the difference in scan duration was analyzed in this subject. Thus, 8 subjects were included and analyzed with respect to quantitative intracardiac measures.

### 4D flow and 2D flow

**Fig. 2:**
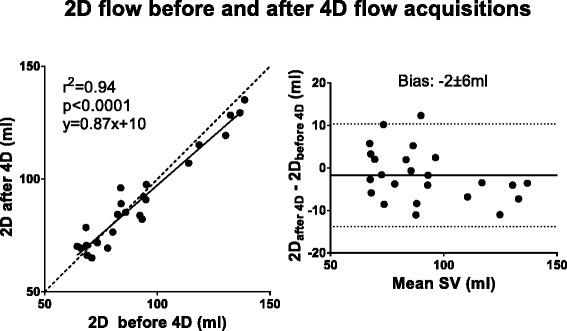
Stability of stroke volume (SV) over the experiment in healthy volunteers. The scatter plot (*left*) shows SV (ml) from 2D flow acquired before and after 4D flow acquisitions. The Bland-Altman analysis (*right*) shows bias (solid line) ±1.96SD (dashed lines). The SV did not vary significantly over the experiment

There was a strong correlation between 2D flow measurements before and after the 4D flow sequences, indicating stability of both physiology and measurement over the scan duration (R^2^ = 0.94, *p* < 0.0001; −1.7 ± 6.2 ml Fig. 2). For comparison between 2D and 4D, the 2D flow acquired prior to 4D was used. Bias is presented in absolute measures and as percentage in Table 3.

**Table 3:** Bias in absolute measures and percentage difference

Comparison	Absolute	Percentage
2D before and after 4D (Fig. 2)	−2 ± 6 ml	−1 ± 7 %
5ch Resp(+) 4D SV vs 2D SV (Fig. 3)	−10 ± 12 ml	−10 ± 11 %
5ch Resp(−) 4D SV vs 2D SV (Fig. 3)	−13 ± 12 ml	−14 ± 12 %
32ch Resp(−) 4D SV vs 2D SV (Fig. 3)	−12 ± 14 ml	−12 ± 13 %
PT Resp(+) vs PT Resp(−) (Fig. 4)	0 ± 9 ml	2 ± 12 %
5ch Resp(+) PT Inflow vs Outflow (Fig. 5)	7 ± 9 ml	8 ± 9 %
5ch Resp(−) PT Inflow vs Outflow (Fig. 5)	13 ± 12 ml	17 ± 16 %
32ch Resp(−) PT Inflow vs Outflow (Fig. 5)	8 ± 11 ml	8 ± 10 %
32ch Resp(−) vs planimetric SV (Fig. 6)	−4 ± 18 ml	−8 ± 23 %
5ch Resp(−) vs 5ch Resp(+) mean KE (Fig. 8)	0.1 ± 0.2 mJ	4 ± 12 %
5ch Resp(−) vs 5ch Resp(+) KE peak values (Fig. 8)	0.1 ± 0.8 mJ	1 ± 17 %
Vortex volume Resp(−) vs Resp(+) (Fig. 9)	−3 ± 9 ml	−4 ± 13 %
4D SV 32ch Resp(−) vs 5ch Resp(−) (Fig. 10)	2 ± 7 ml	2 ± 8 %

**Fig. 3:**
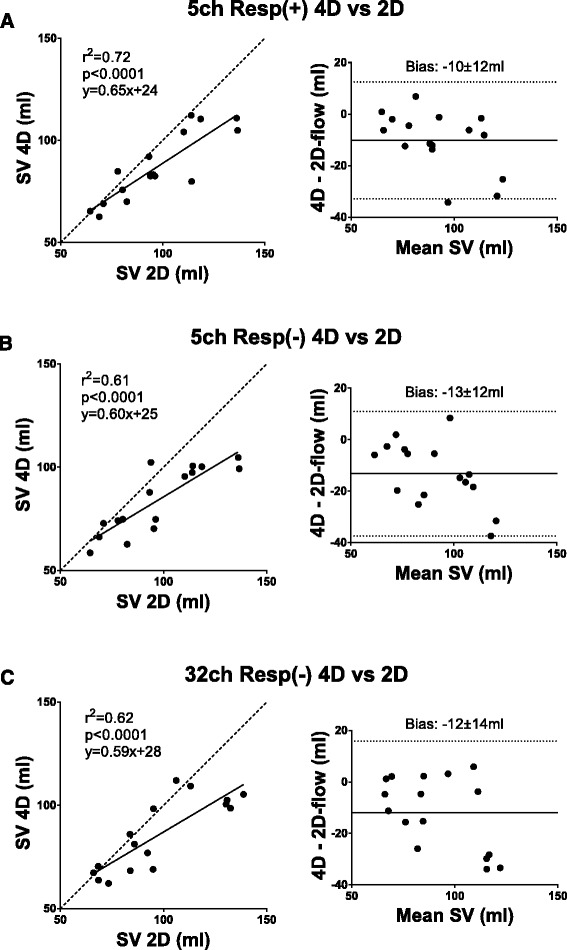
Comparison between SV from respiratory-gated 4D (Resp(+)) and non-gated 4D (Resp(−)) vs 2D flow. Results for healthy volunteers. Both SV deduced from Ao and MPA flow are included. The left column shows scatter plots of SV from 4D flow (ml) and 2D flow (ml). Dashed lines show the line of identity. The right column shows the corresponding Bland Altman plots (bias ± 1.96SD). Panel **a** shows comparison between Resp(+) 4D and 2D. using a 5 channel coil. Panels **b** and **c** show the same results for SV from 4D Resp(−) acquired with a 5 channel cardiac coil and a 32 channel cardiac coil, respectively. The bias and spread were similar for all three methods

Stroke volume (SV) in aorta and pulmonary artery with 4D flow was lower compared to SV from 2D flow (5ch Resp(+): 88 ± 18 ml vs 97 ± 24 ml, *p* < 0.01; 5ch Resp(−): 86 ± 16 ml vs 97 ± 24 ml, *p* < 0.01). Regression analysis showed somewhat higher correlation between SV from 4D flow and 2D flow for Resp(+) compared to the Resp(−) sequences, and Bland-Altman analyses showed similar bias between the different 4D sequences and 2D flow (Fig. 3). There was a good correlation between SV from 4D Resp(+) and 4D Resp(−) (R2 = 0.55, *p* < 0.001, bias: 2 ± 11 ml).

### Particle tracing analysis in healthy volunteers and in patients

**Fig. 4:**
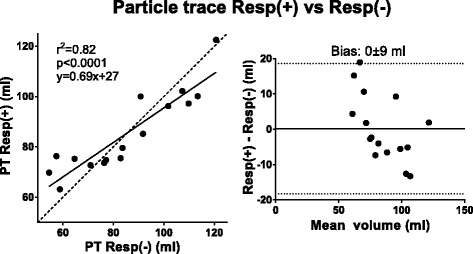
Particle-trace analysis in healthy volunteers. Scatter plot with particle trace (PT) derived SV (ml) from 5ch Resp(+) vs 5ch Resp(−). Dashed line represent line of identity. Bland-Altman analysis shows the bias (bias ± 1.96SD)

**Fig. 5:**
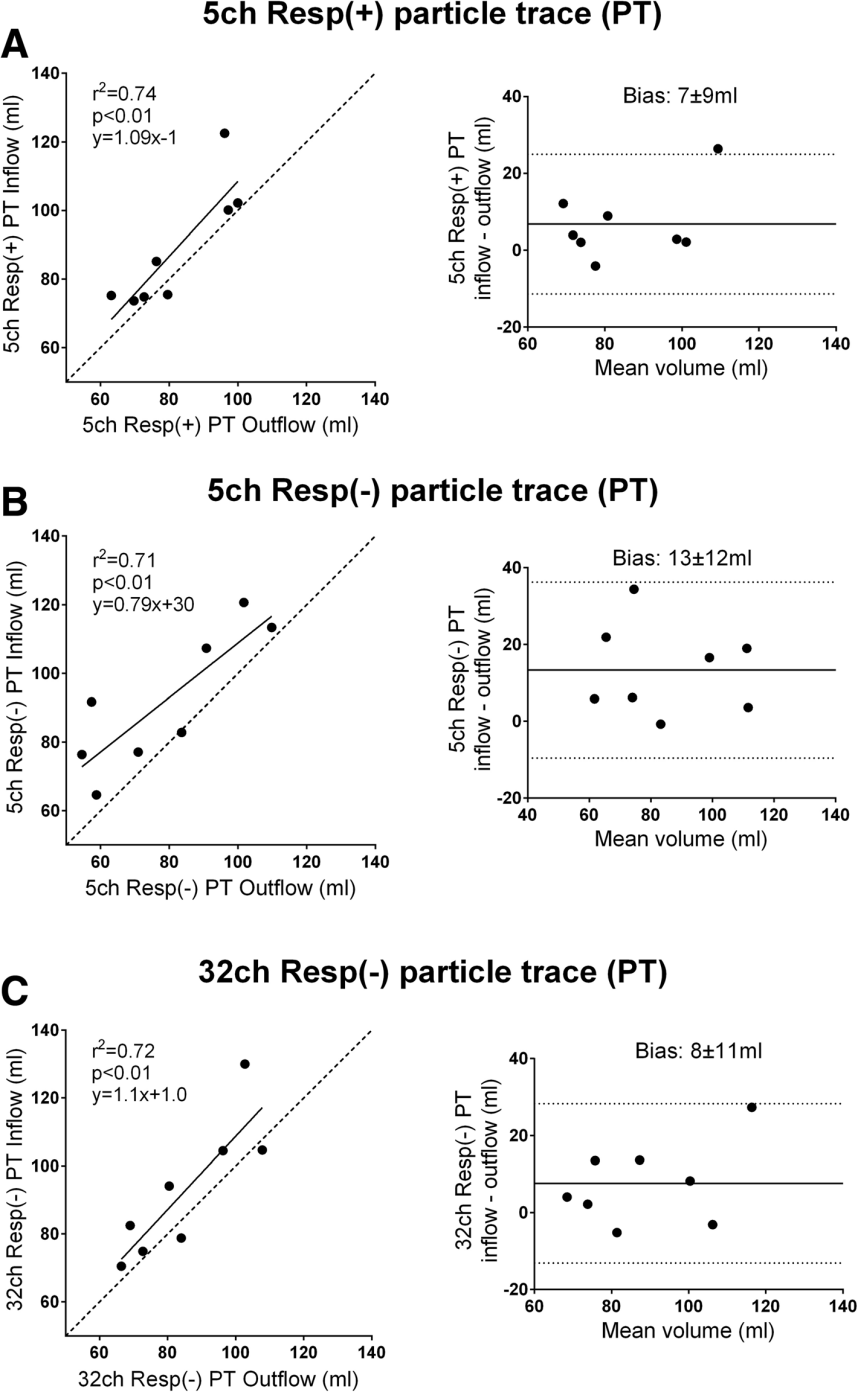
Comparison between inflow vs outflow in healthy volunteers. There was a similar correlation between 5ch Resp(+) (panel **a**), 5ch Resp(−) (panel **b**), and 32ch Resp(−) (panel **c**). Bias is shown in the corresponding Bland-Altman plot (bias ± 1.96SD)

**Fig. 6:**
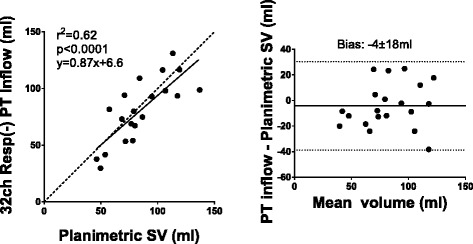
Comparison between particle-trace inflowing blood to planimetric stroke volume in 20 patients with heart failure. Left: the dashed line represents line of identity. Bias is shown in the Bland-Altman plot to the right (bias ± 1.96SD)

**Fig. 7:**
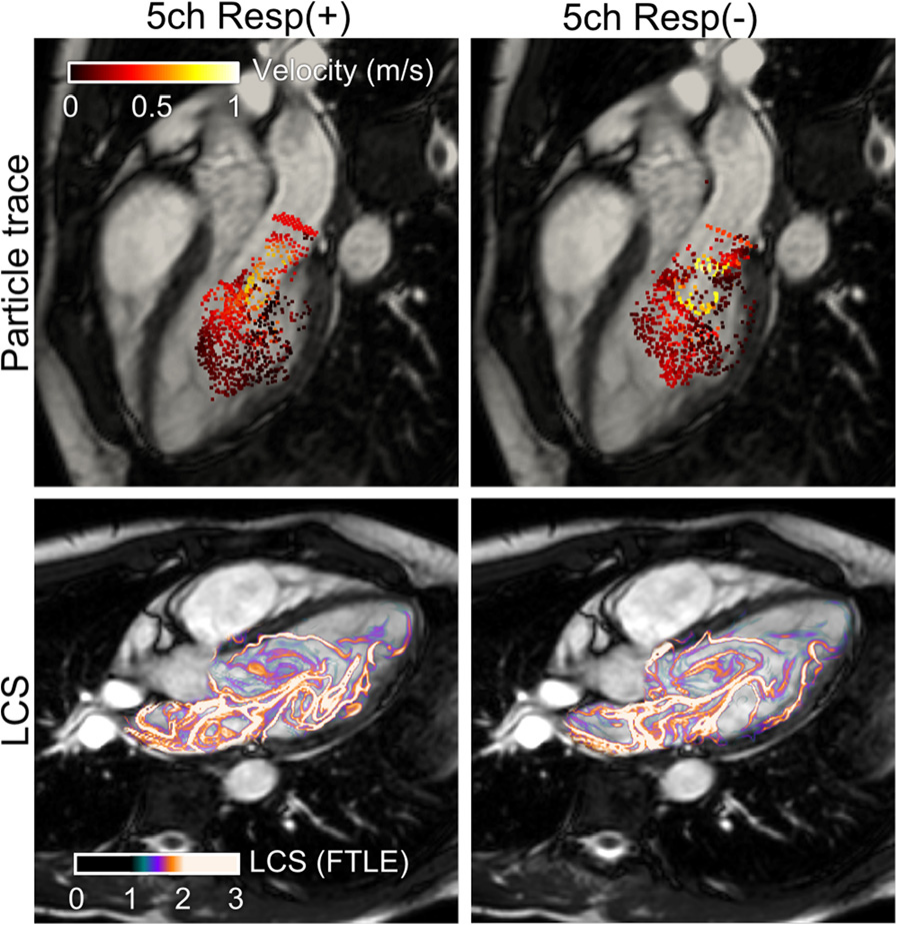
Visualization of particle trace (*top* panels) and vortex volume with LCS (*bottom* panels) in one healthy volunteer using 5ch Resp(+) (*left* column) and 5ch Resp(−) (*right* column) in the 3 chamber view of the heart. For visualizations for all subjects, see Additional file 1

There was good correlation between PT volumes derived from Resp(+) 4D flow compared to PT volumes derived from Resp(−) 4D flow (Fig. 4). PT inflow was higher than PT outflow with 5ch Resp(−) (91.8 ± 20.0 vs 78.4 ± 21.3, *p* < 0.05) but no difference was seen with 5ch Resp(+) or 32ch Resp(−) (88.7 ± 17.9 ml vs 81.9 ± 14.1 ml, *p* = 0.08; and 92.5 ± 19.9 vs 84.9 ± 15.7, p = 0.11). There was no difference in bias between the 5ch Resp(+) and 5ch Resp(−) sequences (6.8 ± 9.3 ml vs 13.3 ± 11.7 ml; *p* = 0.25). Fig. 5 shows correlation and bias ± SD for PT inflow vs outflow. In patients, there was a good correlation between PT inflow volume and planimetric SV (R^2^ = 0.62, −4.2 ± 17.6 ml, Fig. 6) and in healthy volunteers the correlation was good for 5ch Resp(+) (R^2^ = 0.89, 10.7 ± 7.2 ml), 5ch Resp(−) (R^2^ = 0.93, −7.5 ± 5.5 ml), and 32ch Resp(−) (R^2^ = 0.76, −15.0 ± 12.3 ml). Visualization of Resp(+) and Resp(−) particle trace in one healthy volunteer is presented in Fig. 7. Particle trace visualizations for all subjects are shown in Additional file 1.

### Kinetic energy and vortex ring size

**Fig. 8:**
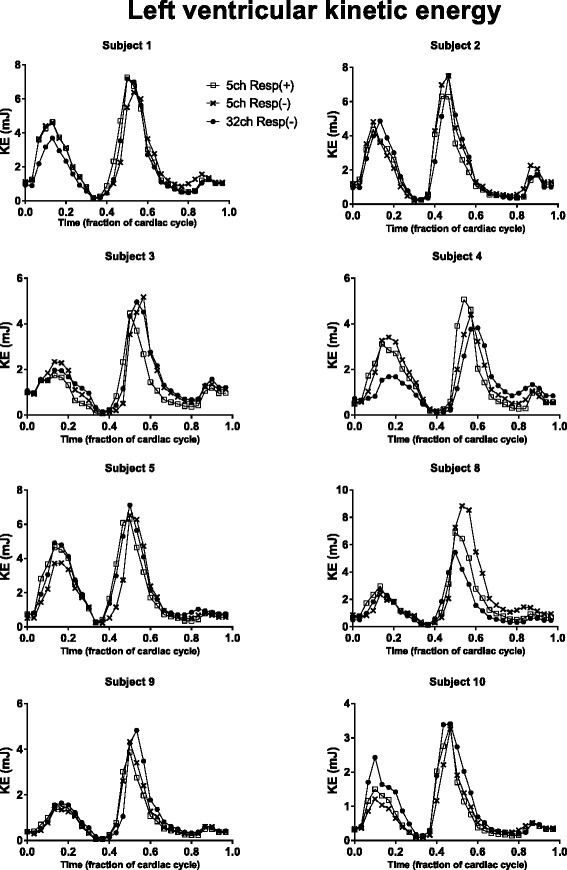
LV kinetic energy (KE, mJ) presented subject by subject over the cardiac cycle as fraction of cardiac cycle in healthy volunteers. Note the similarity of the KE plots for each subject. Subject 8 performed the 32ch acquisition 36 days after the 5ch acquisition, which may affect the appearance of the KE curve

**Fig. 9:**
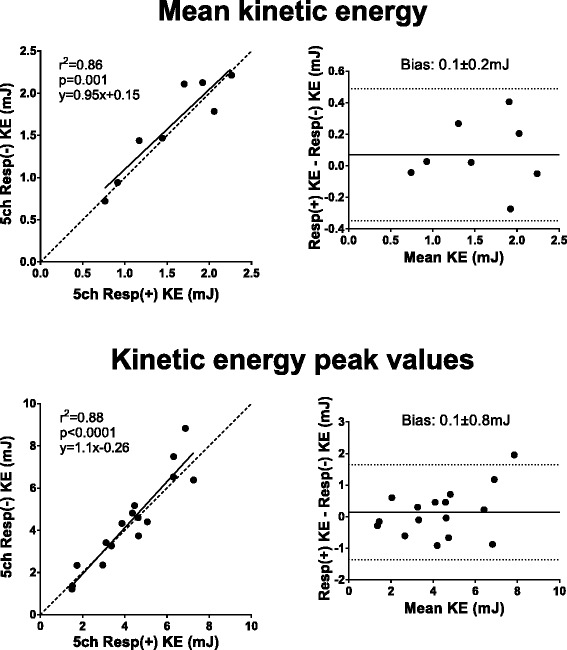
Mean LV kinetic energy (KE, mJ) and peak KE values for 5ch Resp(−) vs 5ch Resp(+) in healthy volunteers. Note the significant correlation between the two sequences. Dashed lines represent line of identity. The bias is shown in the corresponding Bland-Altman plot (bias ± 1.96SD)

**Fig. 10:**
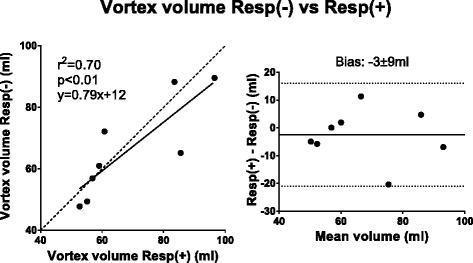
Vortex-volume results for 5ch Resp(−) compared to 5ch Resp(+) in healthy volunteers. There was a statistically significant correlation. Dashed line represents line of identity. Bland-Altman analysis shows the bias (bias ± 1.96SD)

Kinetic energy was studied on individual basis in the healthy volunteers and the KE plots are presented subject by subject in Fig. 8. There was good correlation between Resp(+) and Resp(−) mean KE values (R^2^ = 0.86, *p* = 0.001; bias 0.07 ± 0.21 mJ) and peak KE values (R^2^ = 0.88, *p* < 0.0001; bias 0.14 ± 0.77 mJ) (Fig. 9). For vortex volume, there was good correlation between 5ch Resp(+) and 5ch Resp(−) (R^2^ = 0.70, *p* < 0.01; bias −2.5 ± 9.4 ml, Fig. 10). Visualization of Resp(+) and Resp(−) vortex ring delineation in one healthy volunteer is presented in Fig. 7. Vortex ring visualizations for all subjects are shown in Additional file 1.

### Comparison between coils

**Fig. 11:**
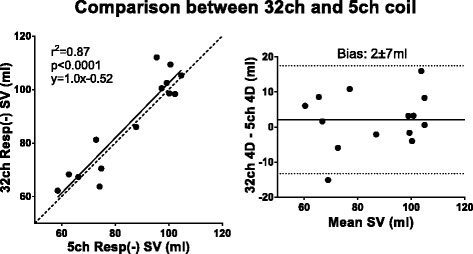
Comparison between 32ch and 5ch coil in healthy volunteers. There was a strong correlation between 4D flow SV using 32ch and 5ch coil. Dashed line represents line of identity. Bland-Altman analysis shows the bias (bias ± 1.96SD)

There was no statistically significant difference between 32ch Resp(−) and 5ch Resp(−) regarding 2D flow (96 ± 25 ml vs 97 ± 24, *p* = 0.68; bias 1.6 ± 9.5 ml), 4D SV (88 ± 18 ml vs 86 ± 16 ml, *p* = 0.32; bias 2.1 ± 6.7 ml, Fig. 11), particle trace inflow (94 ± 19 ml vs 92 ± 20 ml, *p* = 0.38; bias 2.7 ± 8.3 ml), particle trace outflow (85 ± 16 ml vs 81 ± 19 ml, p = 0.47; bias 3.6 ± 7.0 ml), mean KE (1.6 ± 0.4 mJ vs 1.5 ± 0.5 mJ, p = 0.69; bias 0.04 ± 0.25 mJ), and vortex-ring size (70 ± 14 vs 65 ± 16, *p* = 0.38; bias 4.7 ± 11.8 ml). Also, KE curves from 32ch acquisition were similar to 5ch Resp(−) and 5ch Resp(+) (Fig. 8).

### Scan duration

In three healthy volunteers scan duration was not measured and could not be assessed retrospectively. Thus, scan duration was measured in seven subjects. Paired non-parametric analysis (Wilcoxon) of the 5ch data shows a consistently shorter scan duration for the Resp(−) sequence compared to Resp(+) (27 ± 9 min vs 61 ± 19 min, *p* < 0.05).

## Discussion

This study shows a head-to-head comparison and validation of intracardiac flow measures using 4D-flow sequences with and without respiratory gating. Our results show that 4D flow without respiratory gating, Resp(−), yields similar quantitative results and reduced scan time compared to 4D flow with respiratory gating, Resp(+), and comparable results in patients with heart failure.

### Relation to earlier studies

The results of the present study show that respiratory gating may be excluded when studying intracardiac and large vessel blood flows acquired using 4D flow at 1.5 T. This is in line with results presented by Nordmeyer et al. [20] who showed that the respiratory gating may be omitted at 3 T when studying large vessel blood flow. However, our study at 1.5 T shows a slightly higher bias which can be explained by the field-strength difference as shown in a previous study [7]. Furthermore, we performed a validation between 5ch and 32ch acquired data which shows that 32ch Resp(−) yields similar results as 5ch Resp(−). To our knowledge there is no previous head-to-head comparison between the 32ch and 5ch coil.

This study shows an underestimation of 4D flow SV compared to 2D flow, which is in line with previous results at 1.5 T and 3 T [7, 24]. In a phantom study with steady flow (as opposed to the pulsatile flow in the heart), Brix et al. showed a 10 % overestimation of 4D flow compared to 2D [25], although their in vivo results show a small underestimation (−2 ± 15 ml, computed from presented values). Furthermore, we observed an underestimation of particle trace volumes compared to 2D flow, which is in line with previous studies on particle tracing [5, 12]. This may be explained by the clinically non-significant mitral regurgitation commonly seen in healthy volunteers [26] when comparing LV planimetry to 2D aortic blood flow. Further error sources include low temporal and spatial resolution, which have been shown in a computational study to give lower particle tracing volumes [27]. A mild mitral insufficiency can also explain the larger particle trace inflow volume compared to particle trace outflow volume seen in this study.

In this study we observed the effect of acquiring 4D flow without respiratory navigator. When using Resp(−), scan time was reduced by 58 %. This can be compared to the 40 % reduction seen in the study by Nordmeyer et al. [20]. The difference in scan time reduction between our study and that of Nordmeyer et al. may be explained by a multitude of factors including protocol design, differences in positioning and size of acceptance windows of the respiratory navigator, and physiological differences in e.g. heart rate and respiratory rate. The scan durations in this study are, even upon omission of the respiratory navigator, longer compared to contemporary work [10, 11, 28]. The scan time is influenced by 1) k-space read-out technique (we use Cartesian in contrast to the faster spiral or radial read-outs [10, 11, 28]), 2) degree of parallel acquisition (we use 2 views per segment compared to up to 8 views per segment), and 3) size of the 3D box covering the area of interest, where we covered the entire heart and included the atria and ascending aorta, compared to others who only acquire data limited to e.g. the left ventricle.

In the present study a VENC of 200 cm/s was used in arteries since the flow in these vessels, in absence of stenosis, often reach 150 cm/s and to avoid aliasing in routine clinical scans. Since anti-aliasing can be cumbersome in the clinical work flow, we chose a high VENC to avoid aliasing in most cases. Intracardiac velocities seldom exceed 100 cm/s, and we therefore chose a lower VENC of 100 cm/s to minimize noise since the velocity noise is proportional to VENC, as shown by Nilsson et al. [29] In the case aliasing occurs, it is possible to perform aliasing corrections in a research setting.

### Clinical implications

When applying respiratory gating, the 4D flow sequence may be extensively prolonged. Gating efficiency has been reported to range from 50–70 % [20, 30], i.e. up to doubled scan duration. In this study, the mean scan duration for Resp(+) was twice as long as Resp(−), and yet the two different techniques yielded similar results for SV when comparing to 2D flow and in PT analyses, and also for whole heart 4D measurements such as KE. This suggests that the 4D flow measures considered in this study can be acquired without respiratory gating.

We chose to analyze the patients with regards to particle-trace inflow vs planimetric stroke volume, since this comparison is not influenced by the presence of mitral insufficiency commonly seen in patients with dilated ventricles. Our results show that 4D Resp(−) can be used with comparable results as in healthy volunteers, and that intracardiac diastolic blood flow can be accurately measured in patients.

Flow in the right atrium (RA) is known to be significantly affected by the respiratory cycle [31]. Therefore, gating the 4D flow acquisition to one point in the respiratory cycle may only show one aspect of RA flow. A 4D flow acquisition without respiratory gating would instead show the average flow pattern in the right atrium, which may better capture physiological RA flow conditions. Our results suggest that this can be performed without affecting measurements of stroke volume and the quantitative parameters in the left ventricle presented in this study.

One subject was excluded due to a low difference in scan times between Resp(+) and Resp(−) acquisitions. This suggests that the respiratory gating accepted almost all data in the Resp(+) scan, and therefore was not effective in discriminating between respiratory phases in this subject. Thus, even when applying careful and accurate positioning of the respiratory navigator, gating may still not work as expected. However, the present results show that for the flow parameters investigated in this study, the effect of respiratory gating is of small importance.

### Limitations

The sample size of the head-to-head comparison in healthy volunteers is small, and therefore non-parametric statistical tests were used. However, the results of the present study are decisive and unlikely to change significantly with a larger sample size. Two subjects underwent CMR with the 5ch and the 32ch coils on two different occasions instead of on the same day. However, this does not affect comparison of the influence of respiratory gating.

## Conclusions

This study shows that 4D flow acquired without respiratory gating yields comparable quantitative measurements, both for vessel flow and for the quantitative intracardiac parameters particle tracing, kinetic energy and vortex ring formation, compared to 4D flow with respiratory gating. Therefore, whole-heart 4D flow without respiratory gating can be used for quantification of intracardiac blood flow.

## Additional file

Additional file 1:
**Visualizations of blood flow using particle tracing and Lagrangian Coherent Structures.**

## Abbreviations

2ch: 2-chamber
3ch: 3-chamber
4ch: 4-chamber
32ch: 32-channel coil
5ch: 5-channel coil
2D: Two-dimensional
4D: Three-dimensional, time-resolved, three-component flow
Ao: Aorta
CMR: Cardiovascular magnetic resonance
CUDA: Compute unified device architecture
ECG: Electrocardiogram
GPU: Graphical processing unit
KE: Kinetic energy
LCS: Lagrangian coherent structures
LV: Left ventricle
MPA: Main pulmonary artery
PT: Particle tracing
RA: Right atrium
Resp(+): Sequence with respiratory gating
Resp(−): Sequence without respiratory gating
ROI: Region of interest
SD: Standard deviation
SV: Stroke volume
TE: Echo time
TR: Repetition time
TFE: Turbo-field echo
VENC: Velocity encoding value
