# Supplementary material for: Whole-heart four-dimensional flow can be acquired with preserved quality without respiratory gating, facilitating clinical use: a head-to-head comparison
Source: BMC Med Imaging. 2015 Jun 18;15:20. doi: 10.1186/s12880-015-0061-4 (PMC4470048; doi:10.1186/s12880-015-0061-4)
Supplement: Additional file 1: — Visualizations of blood flow using particle tracing and Lagrangian Coherent Structures. [file 12880_2015_61_MOESM1_ESM.pdf]

Whole-heart 4D flow can be acquired with preserved quality without respiratory gating facilitating clinical use: Appendix 1

Visualizations of blood flow using particle tracing and Lagrangian Coherent Structures

Parent article

Kanski M, Töger J, Steding-Ehrenborg K, Xanthis C, Bloch KM, Heiberg E, Carlsson M, Arheden H. **Whole-heart four-dimensional flow can be acquired with preserved quality without respiratory gating, facilitating clinical use: a head-to-head comparison.** *BMC Medical Imaging, 2015.*

Contents of this Appendix

This appendix contains visualizations of blood flow in the heart both with respiratory gating (Resp(+)) and without (Resp(-)) in the 4D flow acquisition for 8 healthy volunteers. Figures 1 and 2 show visualizations of vortex ring formation in the left ventricle using Lagrangian Coherent Structures (LCS), and Figures 3 and 4 show visualizations using particle tracing.

Visualizations of LCS were generated using custom software previously described [1]. Particle trace visualizations were generated using FourFlow, open source software for visualization of blood flow (http://fourflow.heiberg.se).

References

1. Töger J, Kanski M, Carlsson M, Kovács SJ, Söderlind G, Arheden H, Heiberg E: **Vortex ring formation in the left ventricle of the heart: analysis by 4D flow MRI and Lagrangian coherent structures.** *Ann Biomed Eng* 2012, **40**:2652–62.

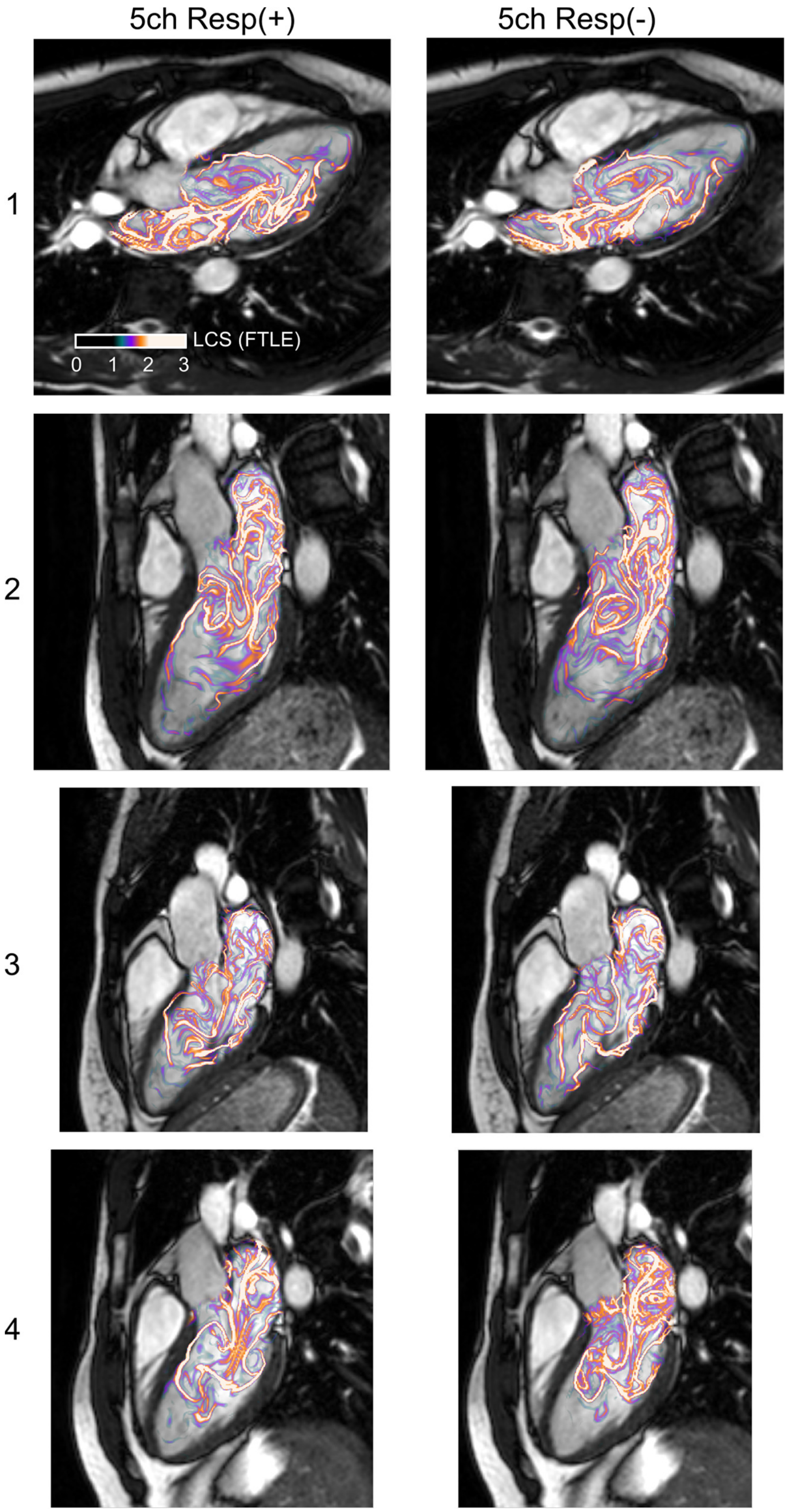

Figure 1: Visualizations of vortex ring formation using Lagrangian Coherent Structures (LCS) in healthy volunteers 1-4 with (Resp(+)) and without (Resp(-)) respiratory navigator.

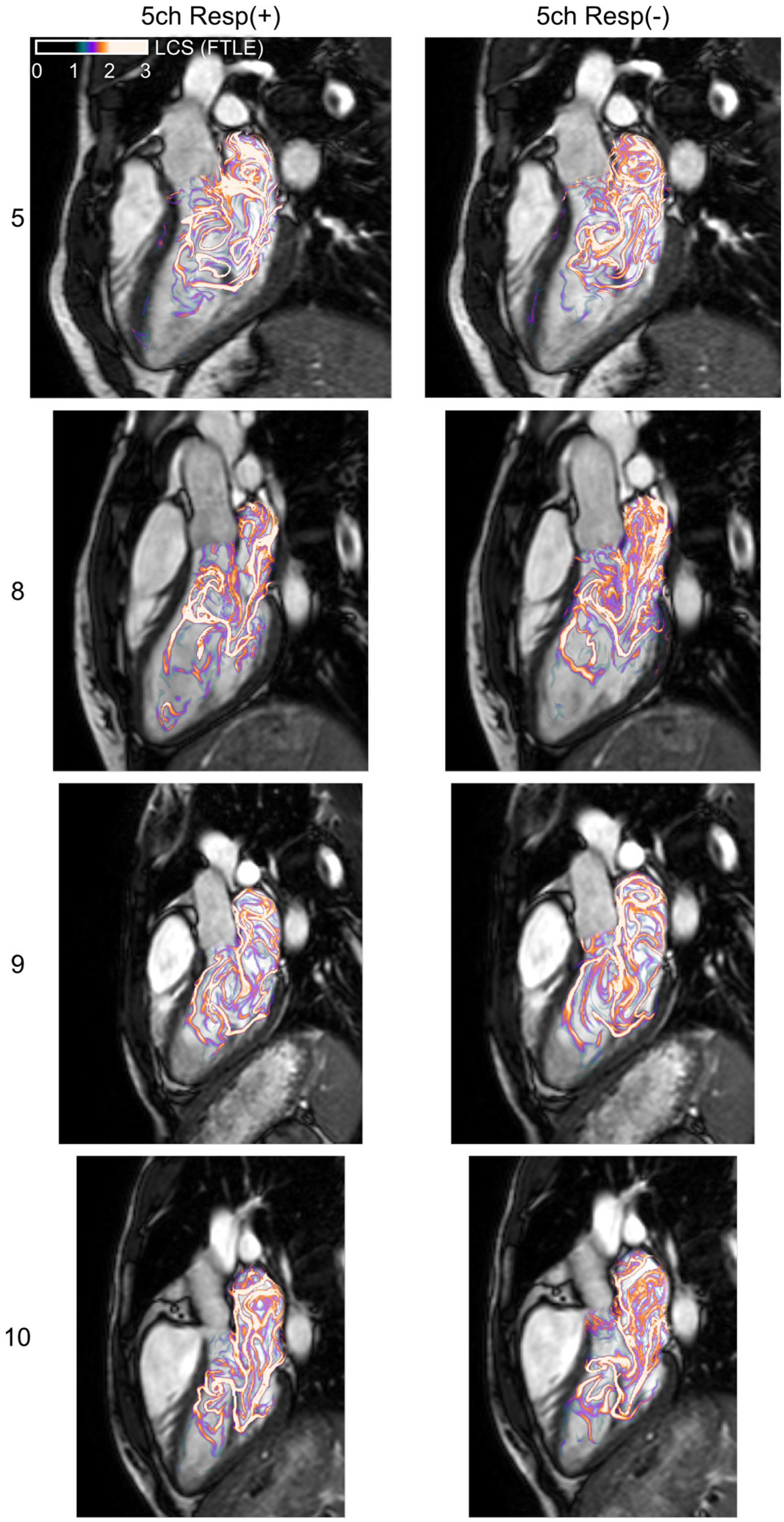

Figure 2: Visualizations of vortex ring formation using Lagrangian Coherent Structures (LCS) in healthy volunteers 5, 8, 9 and 10 with (Resp(+)) and without (Resp(-)) respiratory navigator. Volunteers 6 and 7 were excluded for intracardiac flow measurements due to artifacts in the left ventricle.

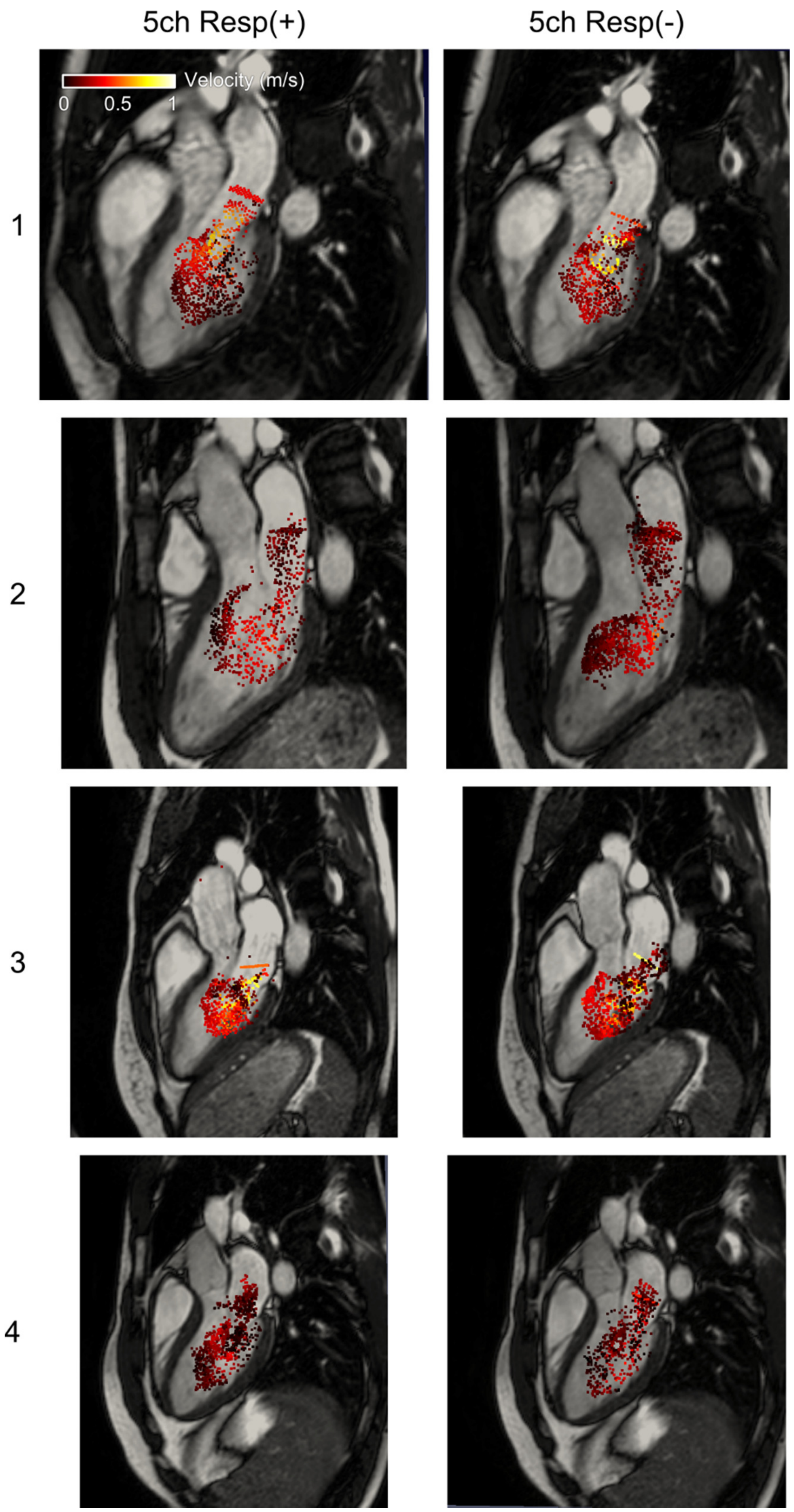

Figure 3: Visualizations of blood flow using particle tracing in healthy volunteers 1-4 with (Resp(+)) and without (Resp(-)) respiratory navigator.

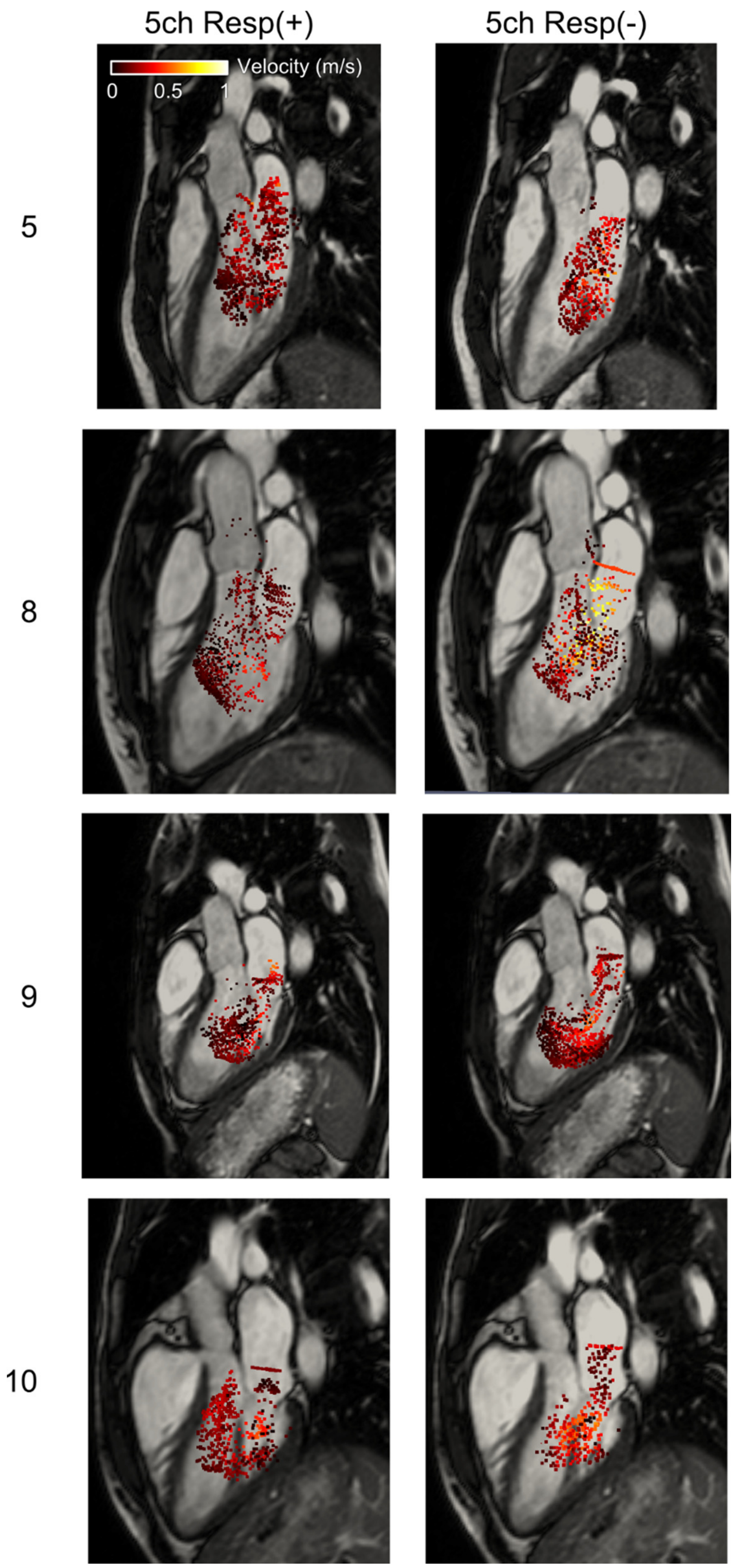

Figure 4: Visualizations of blood flow using particle tracing in healthy volunteers 5, 7, 8 and 9 with (Resp(+)) and without (Resp(-)) navigator. Volunteers 6 and 7 were excluded for intracardiac flow measurements due to RF artifacts in the left ventricle.
